# Spontaneous Reduction of Posterior Shoulder Dislocation Due to Electrical Injury and the Conservative Management of Associated Complications

**DOI:** 10.7759/cureus.78464

**Published:** 2025-02-03

**Authors:** Naif M Alhamam

**Affiliations:** 1 Orthopaedics, King Faisal University, Al-Hofuf, SAU

**Keywords:** complication, conservative approach, electrical injury, posterior dislocation of the shoulder, shoulder

## Abstract

Posterior shoulder dislocation is a rare and often missed injury, usually due to seizures, electrical shocks, or high-impact trauma. A 32-year-old man with a history of diabetes presented to the emergency department following an electric shock that threw him to the ground. He complained of right shoulder pain with limitation of movement. The initial radiographs of both shoulders did not show fracture or dislocation of either shoulder joint, and the condition was managed with an arm sling. A subsequent CT scan confirmed an irregular depression with sclerosis in the subchondral bone in the anterosuperior part of the humeral head, associated with a spontaneous reduction during the course of the electrical injury. He was managed conservatively, and the one-year follow-up showed excellent clinical results. Most shoulder injuries can be treated satisfactorily without surgery, provided there is no significant instability. This case report emphasizes the successful non-surgical treatment of this very rare posterior shoulder dislocation and highlights that careful rehabilitation, along with conservative management, is effective in ensuring significant recovery. This is further supported by evidence seen in the benefits of non-surgical procedures in similar cases.

## Introduction

With an incidence of approximately six per million and a frequency of 2-4% of all shoulder dislocations, posterior shoulder dislocation is an uncommon and often overlooked diagnosis [1,2]. Seizures, electrical shocks, and high-energy trauma are the primary causes. Of these, electrical shocks are responsible for less than 5% of posterior shoulder dislocations [3].

The injury mechanism usually involves a combination of forced internal rotation, flexion, and adduction of the shoulder [4]. Due to its rarity and subtle clinical presentation, posterior shoulder dislocation is frequently missed or delayed in diagnosis, with up to 79% of cases initially being overlooked [5]. Early identification and management require a thorough physical examination and appropriate radiological evaluation.

This report describes a very rare case of posterior shoulder dislocation caused by high-voltage electrical shock, which spontaneously reduced and was conservatively treated, thereby emphasizing early diagnosis and the possibility of conservative treatment.

## Case presentation

A 32-year-old male patient with a past medical history of diabetes was referred to the emergency department following an electric shock sustained while walking on wet ground and coming into contact with a machine. The patient described that this lasted approximately 10 seconds before a companion managed to pull him from the electrical circuit. Upon arrival at the hospital, the team first stabilized the patient, analyzing him for immediate life-threatening injuries. The patient also described shoulder pain subsequent to the shock, which he described as being in the left shoulder. He denied any loss of consciousness during the event but reported dizziness and some confusion immediately following the shock. Further questioning revealed no difficulty in breathing or other neurological symptoms. He described the pain as constant, sharp, and radiating down the posterior aspect of the shoulder. He reported some difficulty with the full range of motion, particularly internal rotation and abduction. On inspection, no obvious deformity was visible; however, tenderness was present over the posterior shoulder joint. The patient could not make overhead movements without considerable discomfort. This was most likely a shoulder dislocation, according to the mechanism of injury and appearance for the initial assessment made by the clinical team. The patient's vital signs remained stable, and the neurological assessment revealed no focal deficits. A detailed physical examination and imaging studies were made, including X-ray, CT, and MRI, to rule out fractures and continue with a detailed assessment of shoulder injury.

**Figure 1:**
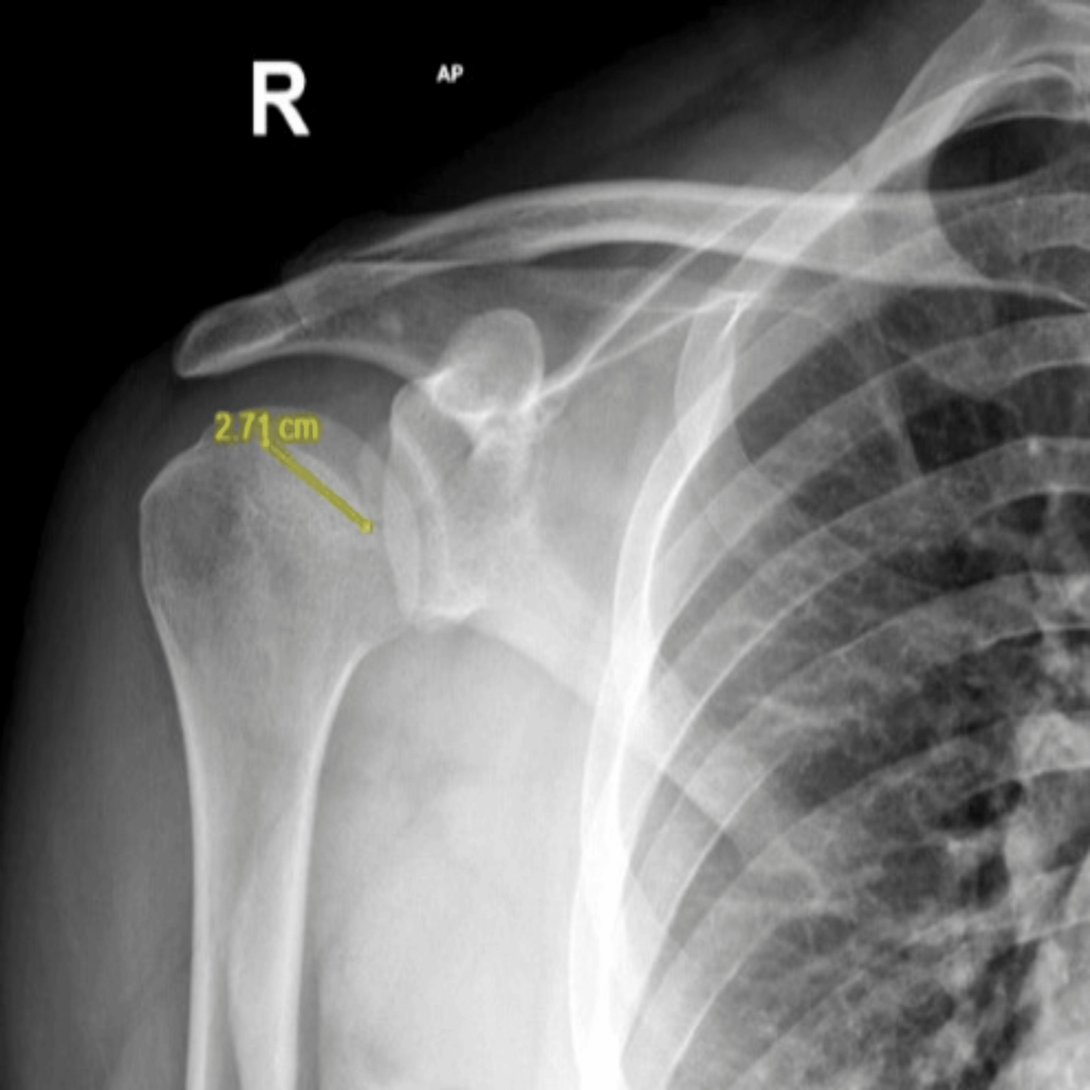
The initial X-ray of the shoulder reveals a depression in the humeral head without any evidence of dislocation or fracture. No associated cortical disruption or malalignment is noted. Therefore, this would indicate an impaction injury at the bone, which may be clinically relevant in shoulder instability.

The patient complained of pain in the right shoulder, with limited movement. The initial radiographic evaluation, including a shoulder X-ray, revealed a depression in the humeral head but did not show evidence of dislocation (Figure 1).

**Figure 2:**
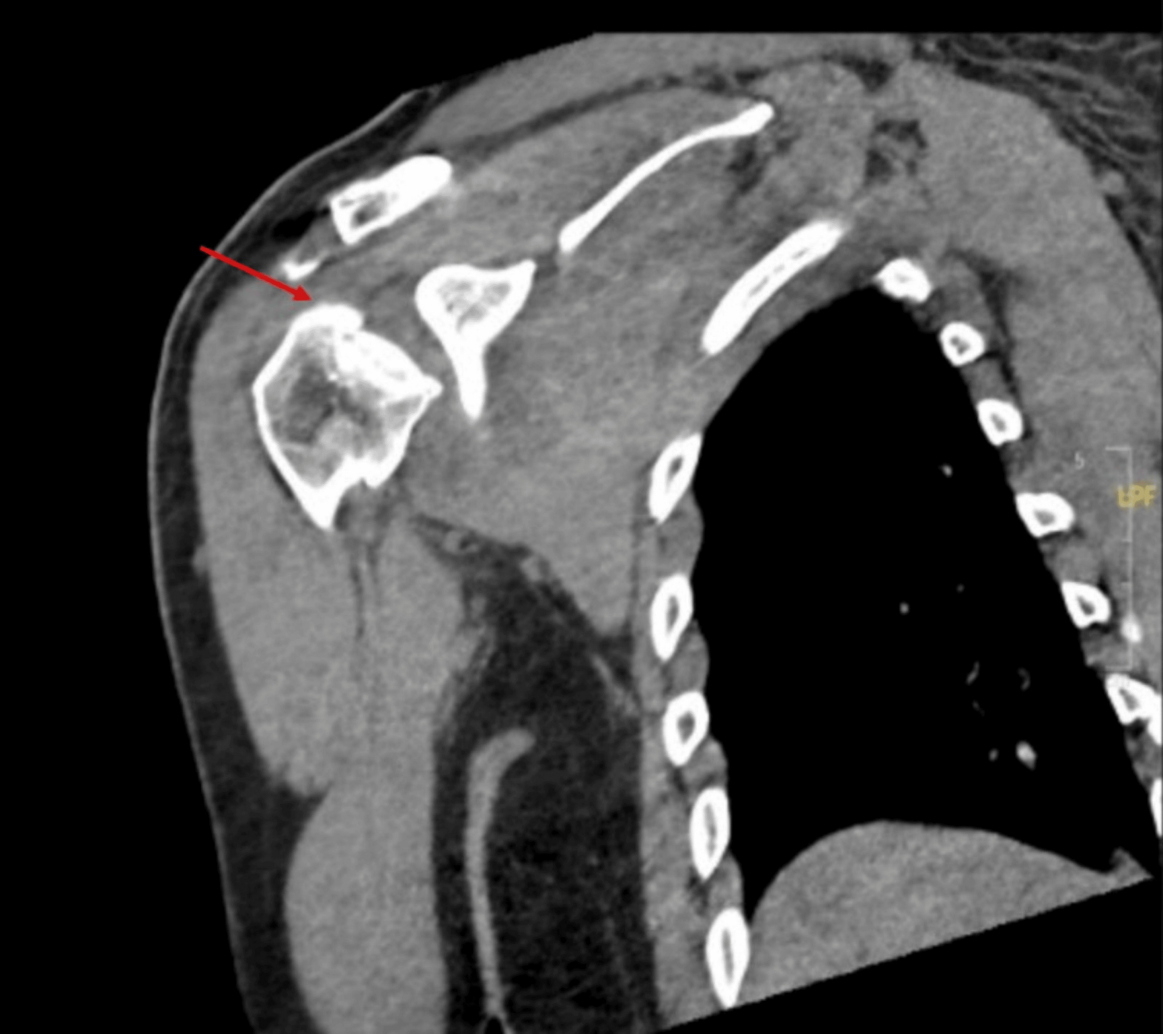
The CT scan of the shoulder shows an irregular depression to the subchondral bone of the anterosuperior humeral head, with the glenohumeral articulation remaining intact. These findings indicate an impaction injury, which might be significant for shoulder stability and clinical management.

An arm sling was applied, and further imaging with a CT scan revealed an irregular depression at the subchondral bone of the anterosuperior aspect of the humeral head, with intact glenohumeral articulation (Figure 2).

**Figure 3:**
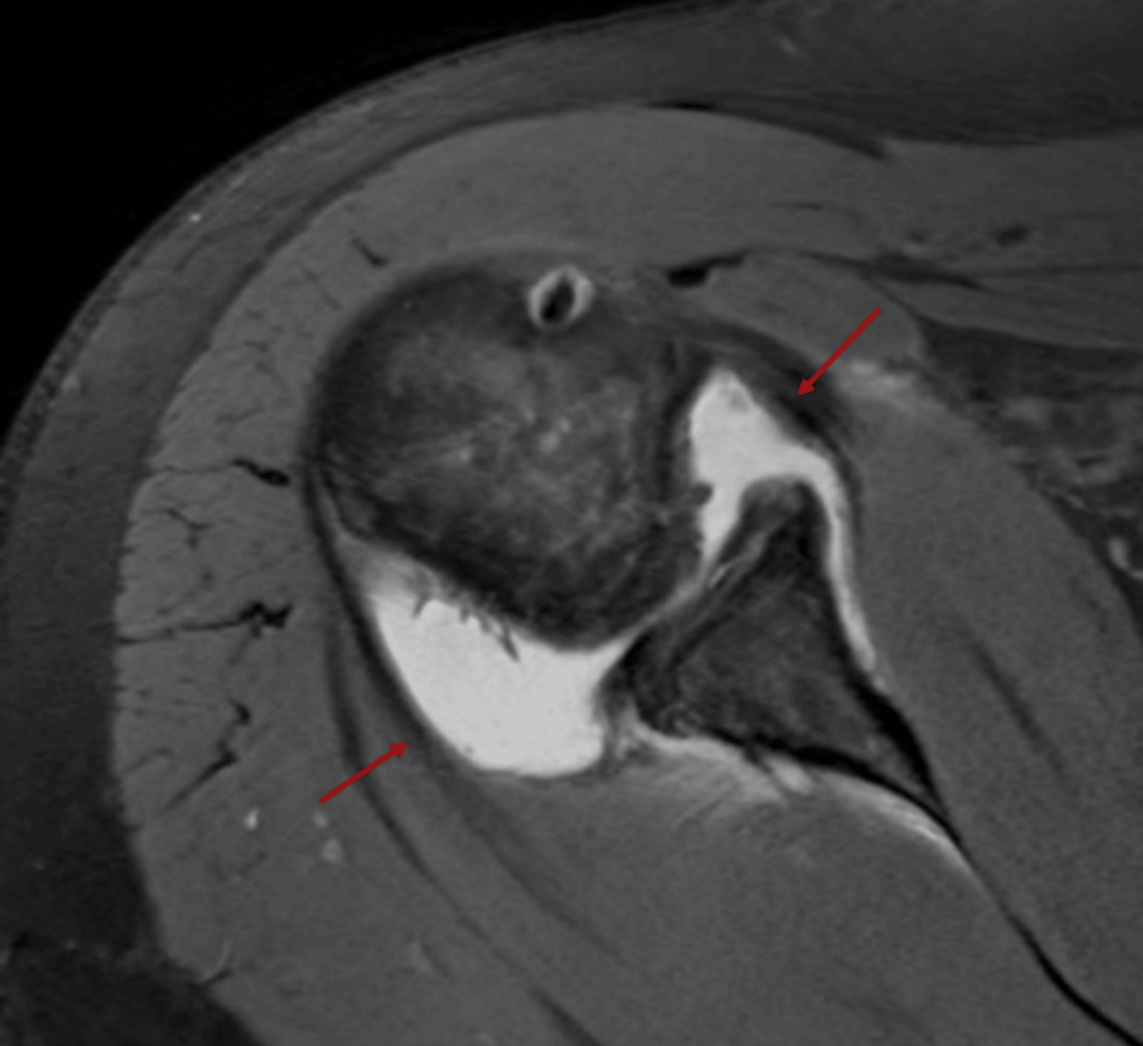
MRI of the shoulder reveals a large glenohumeral joint effusion suggesting spontaneous reduction following a posterior shoulder dislocation during an electrical event. The findings would support a diagnosis of spontaneous reduction of a posterior shoulder dislocation, most likely secondary to an electrical event. There is no associated evidence of a rotator cuff tear or significant labral injury. Features of occult instability of electrically induced shoulder dislocations are shown.

The findings have raised the suspicion of a possible spontaneous reduction of a posterior shoulder dislocation during the electrical event. The patient exhibited improvement post-discharge and continued follow-up in the outpatient clinic. Subsequent MRI findings include soft tissues with a large glenohumeral joint effusion (Figure 3).

**Figure 4:**
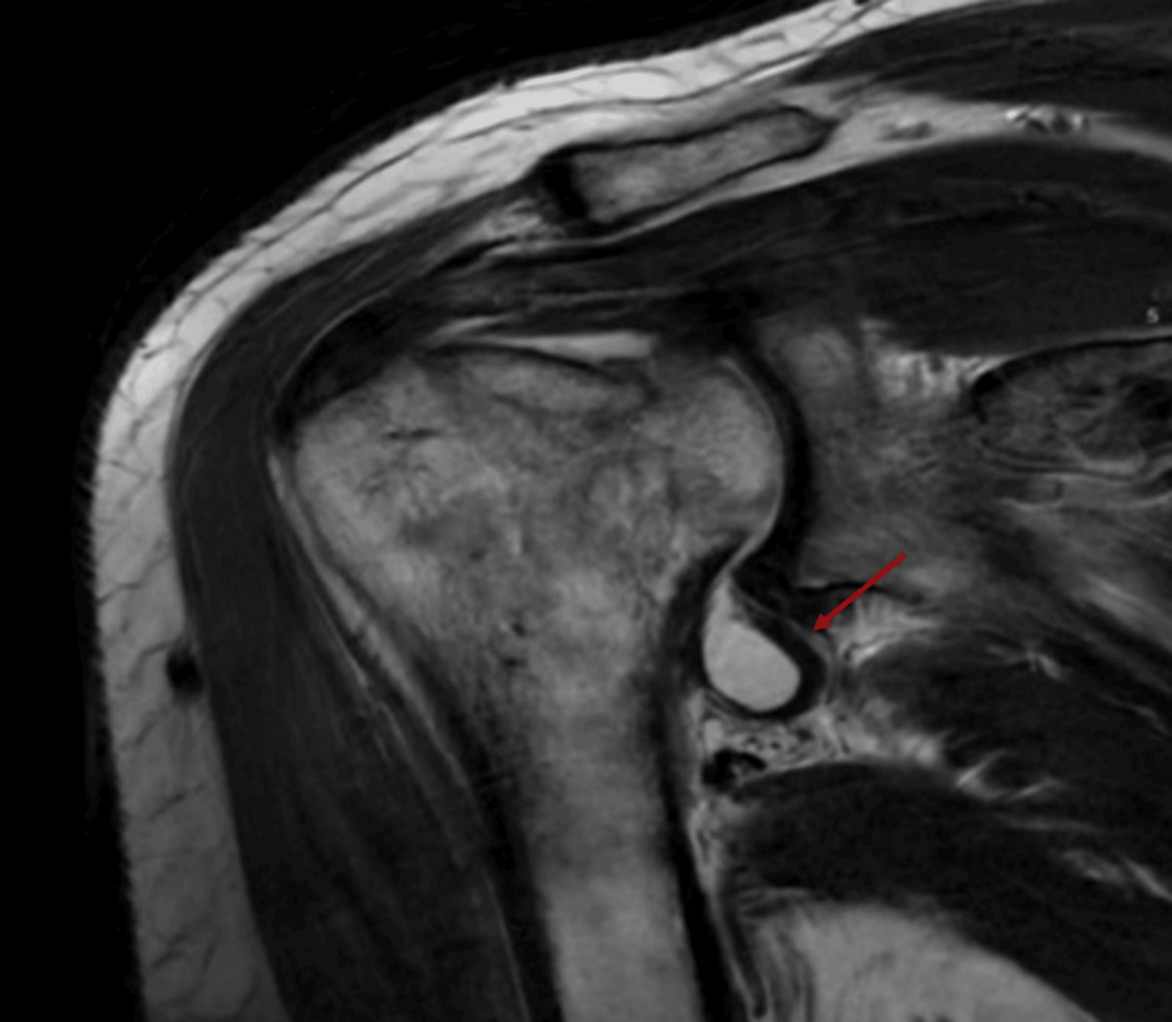
MRI of the shoulder reveals an incomplete inferoanterior to posterior labral tear and reverse Hill-Sachs deformity associated with a previous posterior shoulder dislocation. These findings suggest residual structural changes following posterior instability that may imply implications for shoulder function and future dislocation risk.

There is an incomplete tear of the inferoanterior to the posterior labrum, extending from the 5:00 to the 7:00 position. Concerning the bones and bone marrow, the anterior humeral head compression fracture is consistent, in this case, with the classic reverse Hill-Sachs deformity from the previous posterior shoulder dislocation (Figure 4).

## Discussion

This case illustrates the complex difficulty in managing shoulder injuries due to electrical trauma, specifically in a patient with diabetes mellitus. Imaging modalities showed significant pathologies, including a tear in the labrum, a partial tear in the biceps tendon, and a reverse Hill-Sachs deformity, highlighting both the structural and functional deficits that can occur. Several options for management were discussed with the patient, including arthroscopic repair with concomitant labral repair and McLaughlin procedure for reverse Hill-Sachs deformity, open reduction and internal fixation (ORIF) with the use of an autograft and soft tissue reconstruction, and conservatively with rehabilitation, activity modification, and pain management. Considering the patient's wishes and operability, a conservatively oriented management path was pursued. Over a one-year follow-up period, the patient showed considerable improvements in both shoulder range of motion and functional capacity, supporting the efficacy of non-operative interventions in appropriately considered cases.

The current literature shows surgical intervention to be often preferred in cases of posterior shoulder dislocations with significant Hill-Sachs lesions, recurrent dislocations, and significant soft tissue loss [6]. Despite that, newer studies promote conservatively oriented approaches in cases with no gross instability present. Petkov (2024) has documented that early rehabilitation post-dislocation can result in functional improvement similar to surgical intervention in specific cases [7]. Consistent with such studies, spontaneous reduction, and specifically dislocations secondary to electrical trauma, can stimulate healing without surgical intervention [8]. In addition, comparative studies between surgical and conservatively oriented approaches have proven that, even with a lack of surgical stabilization, function can be similar when conservatively oriented approaches are specifically designed [9]. In the present case, a rehabilitative scheme and careful follow-up facilitated satisfactory improvements, underlining the role of patient preference in decision-making.

Diabetes complicates healing in musculoskeletal tissue, causing delayed healing, increased fibrosis, and prolonged inflammatory processes. These factors can impact therapeutic options, with surgical intervention often resulting in increased complications such as stiffness, infection, and impaired healing of tendons [10]. Where feasible, therapeutic interventions could minimize such complications and allow rehabilitation toward function restoration.

The role of imaging in deciding the therapeutic modality cannot be overestimated. MRI showed significant effusion, suggesting spontaneous reduction, and guided selection for a non-operative intervention. In addition, the CT scan confirmed the presence of a reverse Hill-Sachs lesion in the absence of significant instability, supporting the fact that conservative therapy could act as a safe alternative. Imaging helps in an emerging pool of studies suggesting that not all posterior dislocations require surgical intervention, especially when stability is not compromised.

The case brings out the importance of individualized therapeutic interventions guided by clinic and patient-specific factors. While surgical intervention remains the preferred course of action when instability is observed, this report highlights the effectiveness of conservative therapy in certain cases, particularly when it results in spontaneous reduction and maintains the integrity of stability. There is a necessity for future studies comparing long-term follow-up in relation to therapeutic options in an attempt to maximize protocols for posterior shoulder dislocations [11].

## Conclusions

This case demonstrates that non-surgical management can be effective in shoulder injuries secondary to electrical trauma, even in patients with complicating comorbidities such as diabetes. A significant improvement in the patient's range of motion and function of the shoulder after one year of conservative treatment has underlined the fact that acceptable results can often be achieved with non-surgical intervention, especially when instability is not a concern.

This report draws attention to a tailored treatment approach with the primary choice of non-surgical methods for selected cases and points out an adequate clinical and radiological workup that may form the backbone of the identification of complex patterns of injury, including the so-called spontaneous reductions that might modify the management course. Additionally, this case report adds to the growing evidence for conservative approaches in such scenarios by documenting the successful management of a rare case of posterior shoulder dislocation with associated injuries. Further research and case studies are necessary to refine these strategies and improve patient outcomes, thus enhancing the understanding of rare and challenging presentations of shoulder injuries.
